# BUILDING BRIDGES TO CONNECT BETWEEN NEEDS, RESOURCES, AND RURAL RESIDENTS

**DOI:** 10.1093/geroni/igad104.0516

**Published:** 2023-12-21

**Authors:** Nancy Karlin, Lisa Ann Wiese, Cassandra Ford

## Abstract

The percentage of older adults living in rural areas is predicted to rise with population aging. This impacts access to resources, creates barriers to effective aging, and promotes a unique aging experience. Consequently, demands are growing for services to support healthy aging in rural regions already experiencing healthcare disparities. This symposium will focus on location variations in resource availability, and unique interventions to promote aging in place. In the first and second presentations, researchers report on differences in current service utilization, satisfaction, and perceived future service needs/use among rural versus frontier-residing older adults in Wyoming, and in Nigeria, respectively. In the third presentation, successes and failures of an interdisciplinary approach to increase Alzheimer’s disease and related dementias (ADRD) diagnosis/treatment rates are shared. The fourth group reports the impact of an intergenerational RCT intervention of training older adults in computer literacy and online chair yoga engagement, for decreasing pain levels and cognitive risk. This is a Rural Aging Interest Group Sponsored Symposium.

